# Supplementary material for: Prognostication of chronic disorders of consciousness using brain functional networks and clinical characteristics
Source: eLife. 2018 Aug 14;7:e36173. doi: 10.7554/eLife.36173 (PMC6145856; doi:10.7554/eLife.36173)

# Supplementary file 1:

Some examples of the warped ROIs in the default mode network for one healthy control and three DOC patients with a GOS score 2,3,4, respectively.

(1) "Beijing_750" dataset, patient alias='056', diagnosis = VS/UWS, GOS score=2

- The transformed 4 ROIs and brain network template of DMN. In this figure, the red regions represent the 4 ROIs warped to the patient's fMRI space, whereas the black contours within the brain outline the key brain regions for the warped brain network template of the DMN. Notably, the black contours were only for illumination, and they were obtained above the threshold 10 of the warped brain network template.


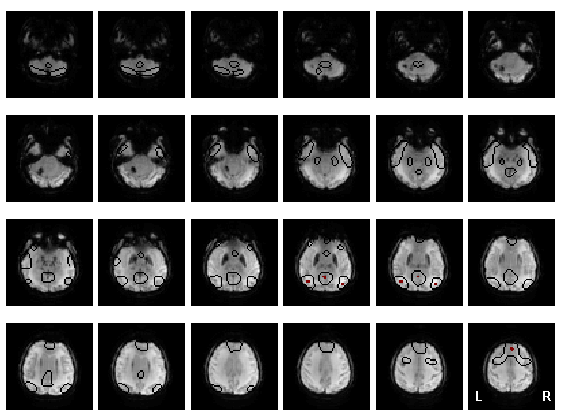


- The functional connectivity Z maps using the warped ROI. The bar in each figure represents the Z value of functional connectivity map of the corresponding ROI across the whole brain.

aMPFC:


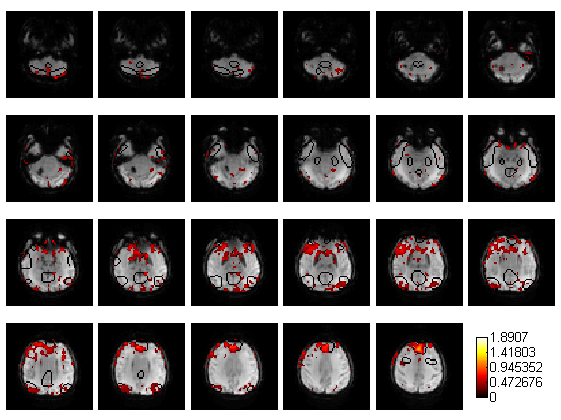


PCC:


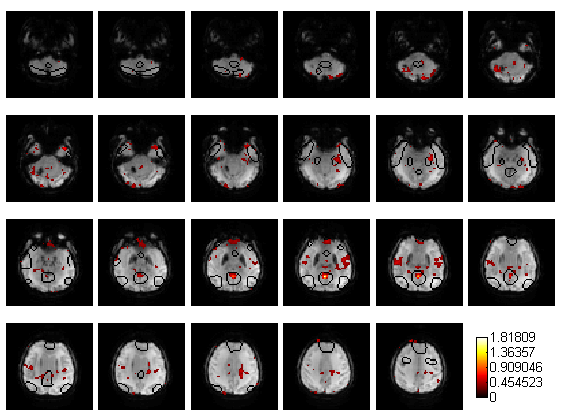


L.LatP:


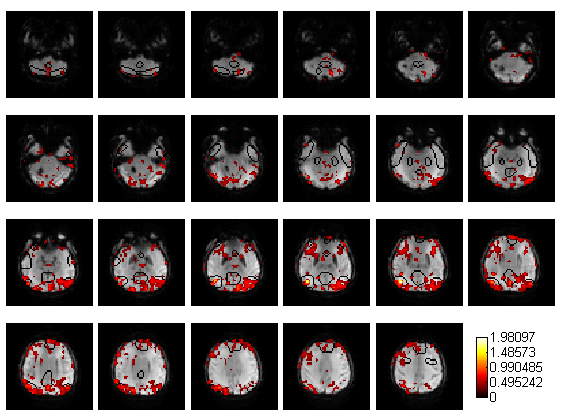


R.LatP:


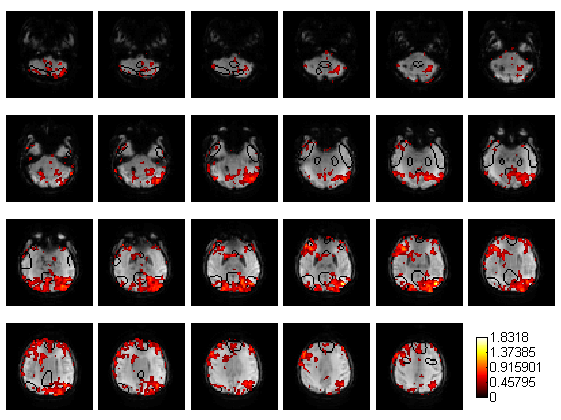


(2) "Beijing_750" dataset, patient alias='013', diagnosis = VS/UWS, GOS score=3

- The transformed 4 ROIs and brain network template of DMN.


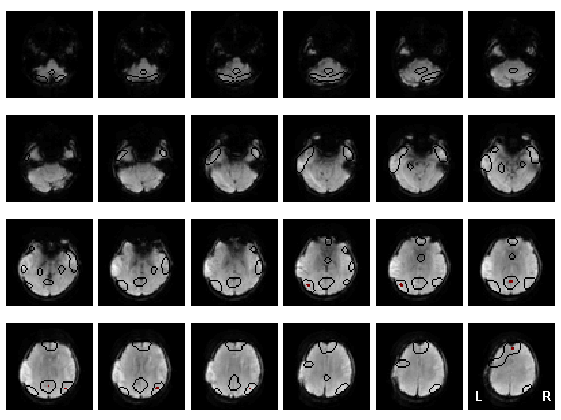


- The functional connectivity Z maps using the warped ROI.

aMPFC:


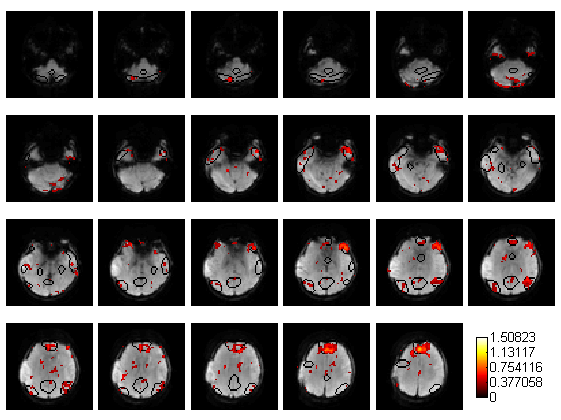


PCC:


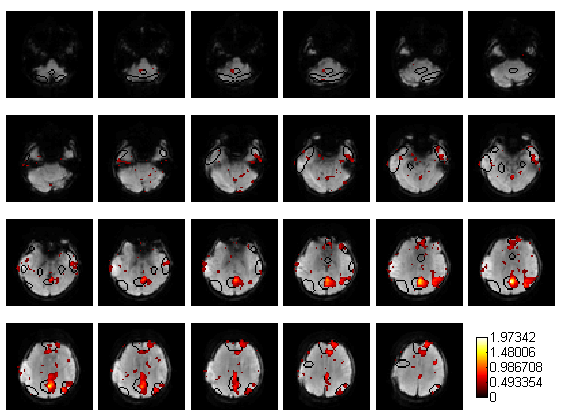


L.LatP:


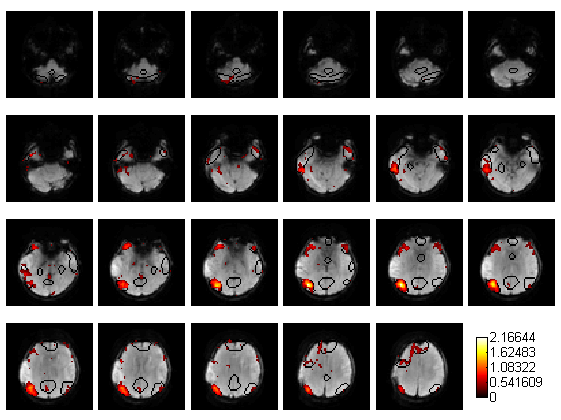


R.LatP:


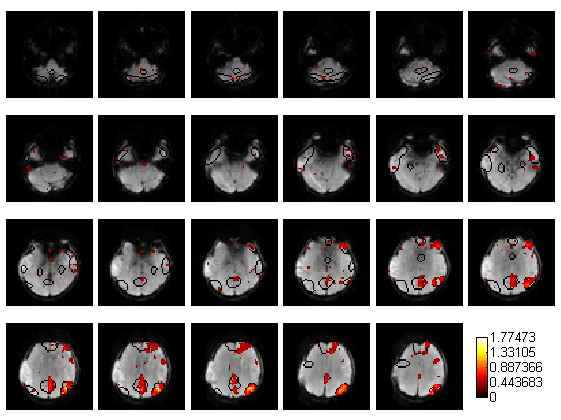


(3) "Beijing_750" dataset, patient alias='003', diagnosis = VS/UWS, GOS score=4

- The transformed 4 ROIs and brain network template of DMN.


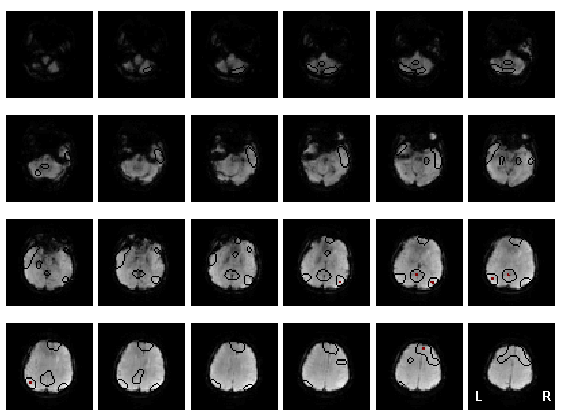


- The functional connectivity Z maps using the warped ROI.

aMPFC:


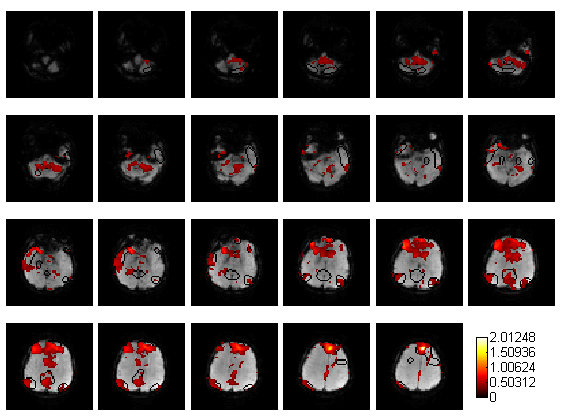


PCC:


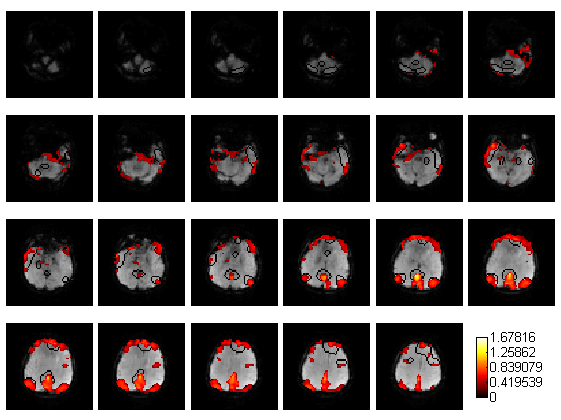


L.LatP:


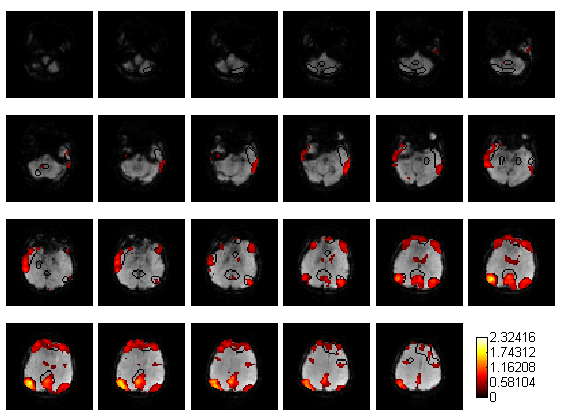


R.LatP:


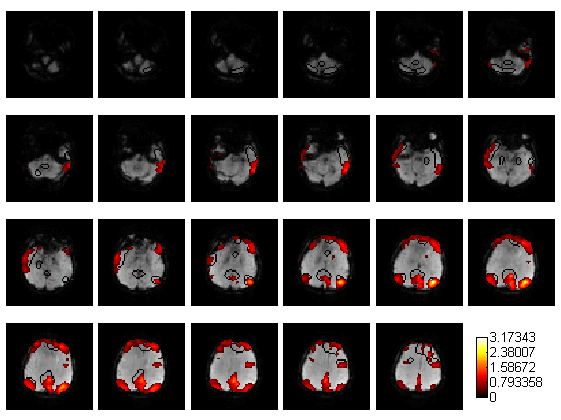


(4) "Beijing_750" dataset, normal control, alias='NC002'

- The transformed 4 ROIs and brain network template of DMN.


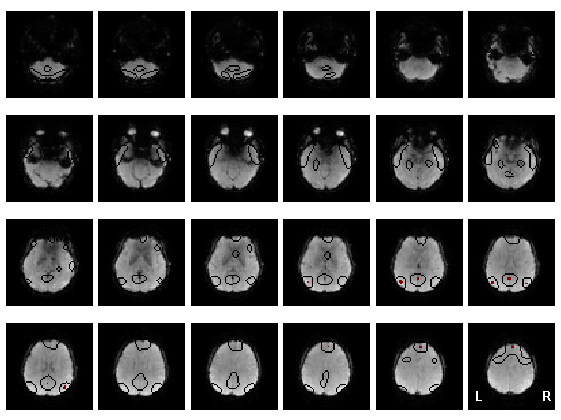


- The functional connectivity Z maps using the warped ROI.

aMPFC:


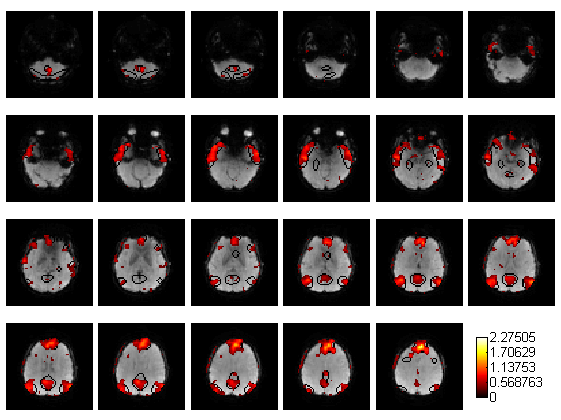


PCC:


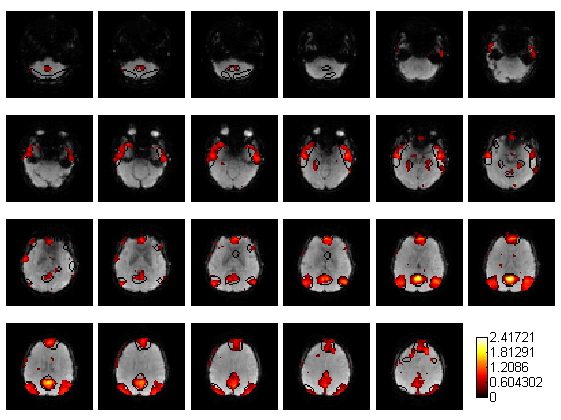


L.LatP:


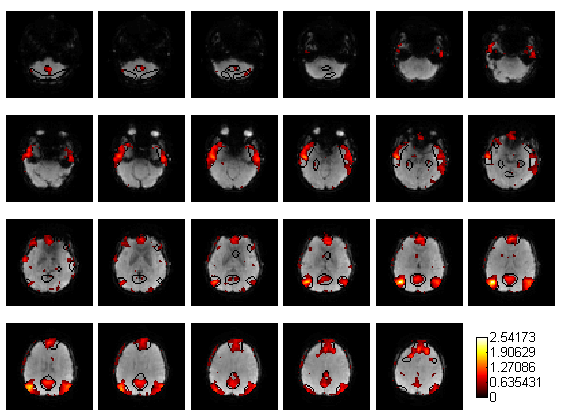


R.LatP:


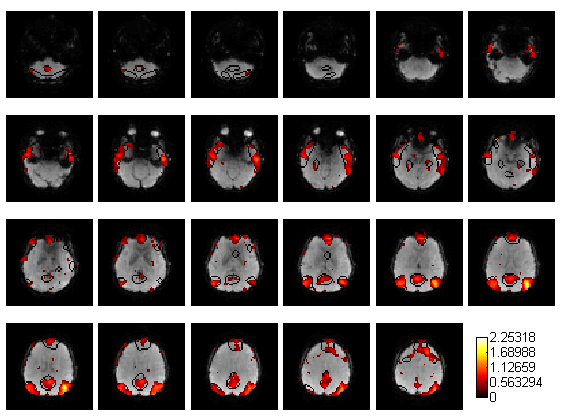

Supplement: Supplementary file 1. [file elife-36173-supp1.docx]
