# Supplementary material for: Prognostication of chronic disorders of consciousness using brain functional networks and clinical characteristics
Source: eLife. 2018 Aug 14;7:e36173. doi: 10.7554/eLife.36173 (PMC6145856; doi:10.7554/eLife.36173)
Supplement: Supplementary file 2. [file elife-36173-supp2.docx]

# Supplementary file 2:

(1). The prognostic regression model using the clinical features alone.


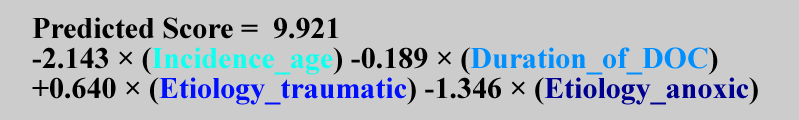


- In the training dataset "Beijing 750", the regression and classification performance were as follows.


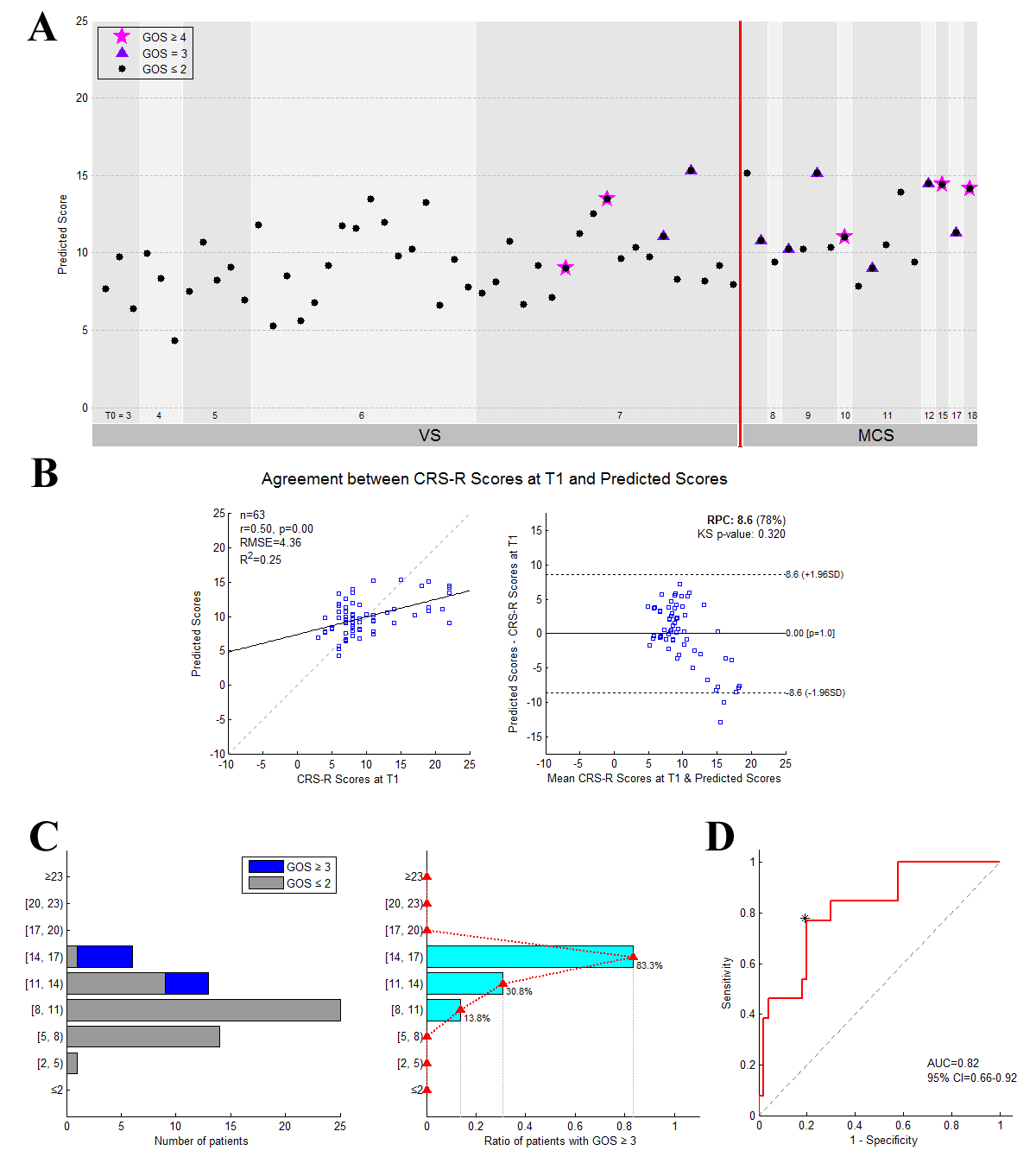


The optimum cut-off value was 10.8. Thus, the classification performance in the training dataset were as follows. ACC = 0.79, Sensitivity = 0.77, Specificity = 0.80, PPV = 0.50, NPV = 0.93, F_1_ score=0.61.

- In the testing datasets "Beijing HDxt" (subplot A) and the testing dataset "Guangzhou HDxt" (subplot B), the regression and classification performance were as follows.


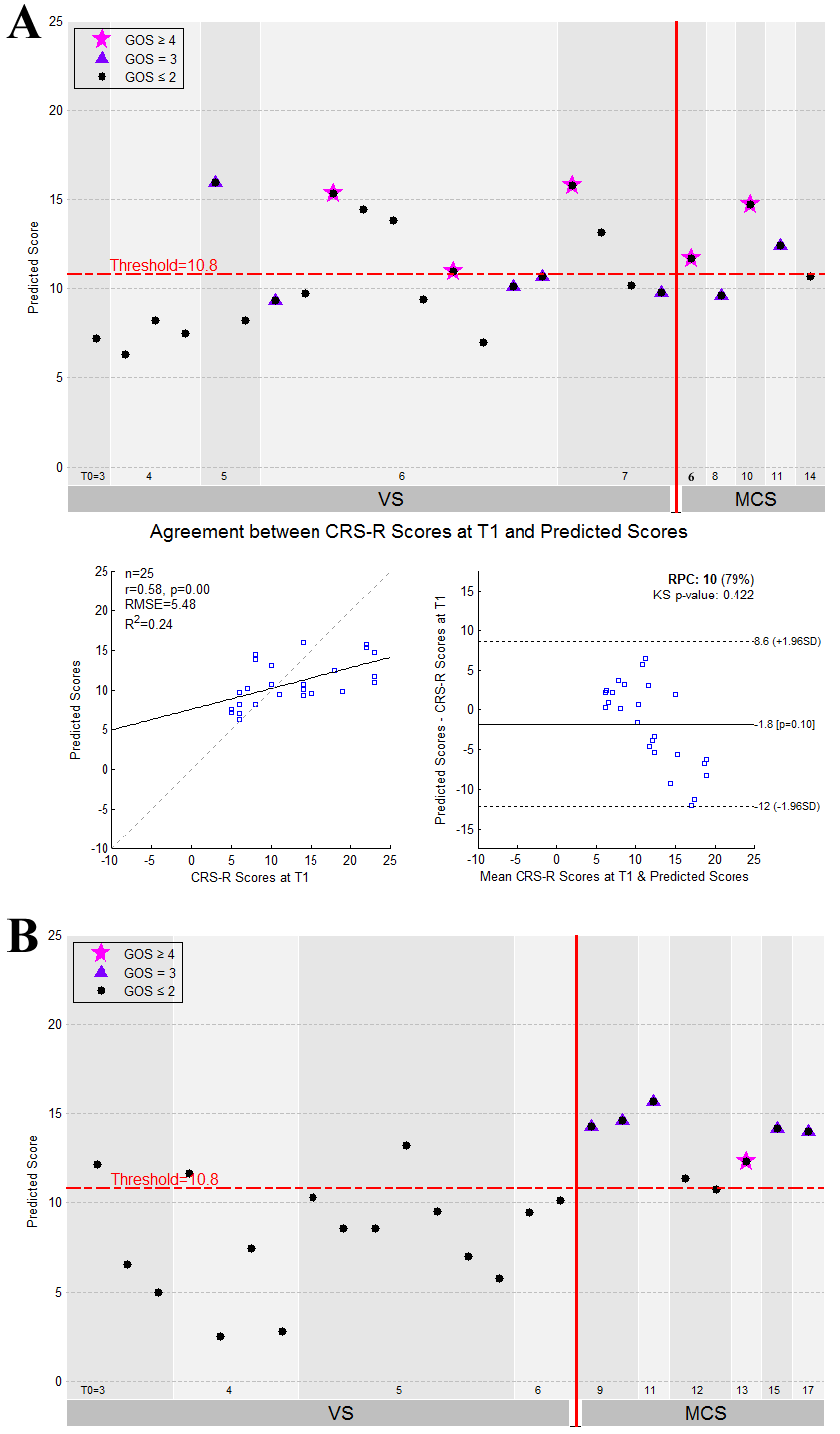


The testing dataset "Beijing HDxt": classification ACC = 0.68, Sensitivity = 0.58, Specificity = 0.77, PPV = 0.70, NPV = 0.67, F_1_ score=0.64; The testing dataset "Guangzhou HDxt" classification ACC = 0.83, Sensitivity = 1.00, Specificity = 0.78, PPV = 0.60, NPV = 1.00, F_1_ score=0.75.

(2). The prognostic regression model using the imaging features alone.


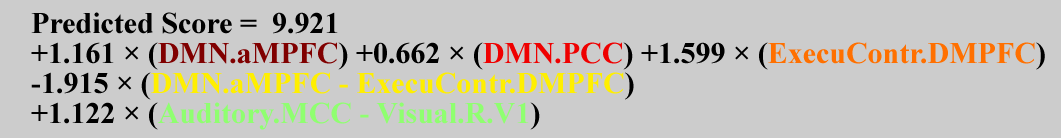


- In the training dataset "Beijing 750", the regression and classification performance were as follows.


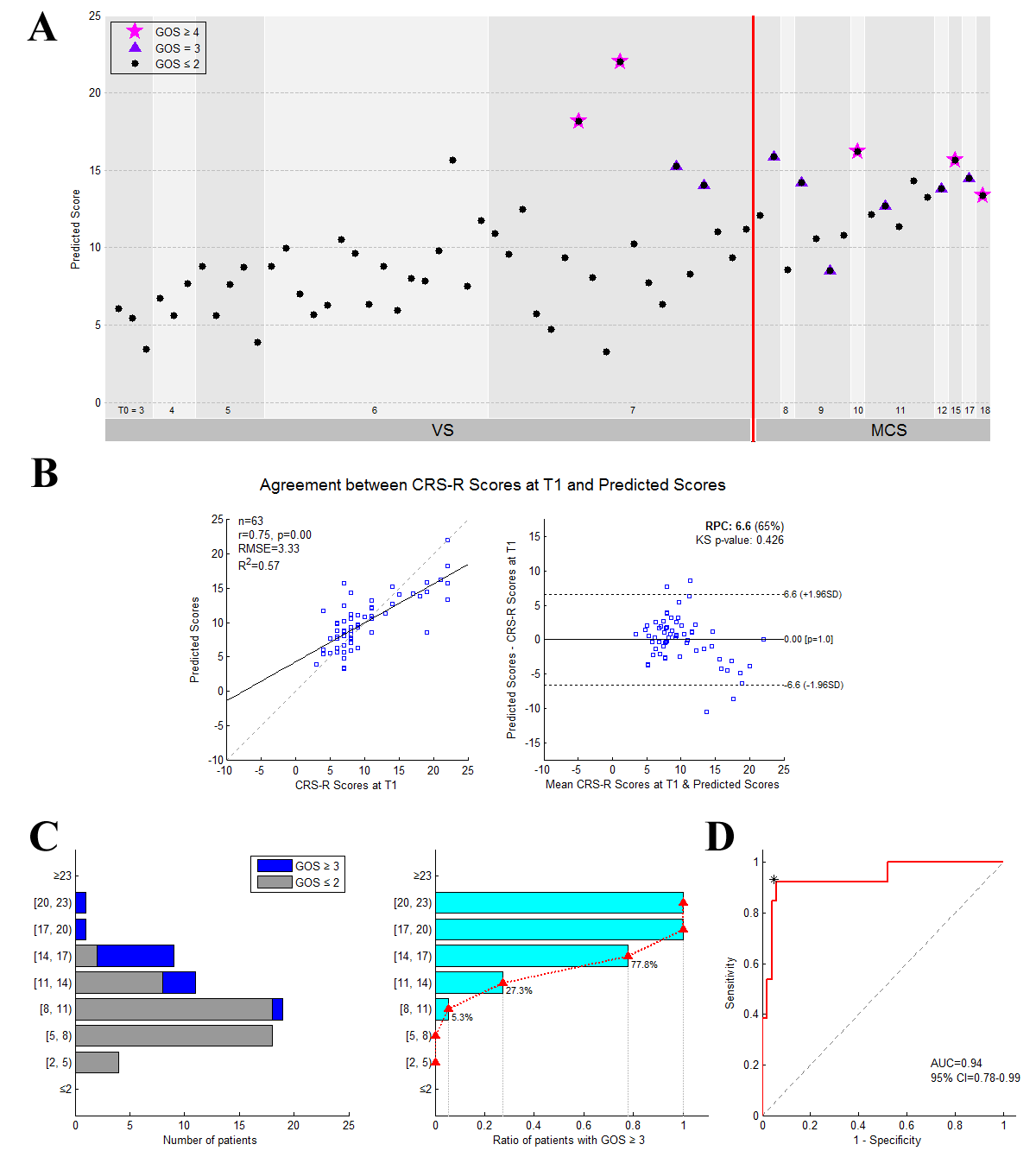


The optimum cut-off value was 12.7. Thus, the classification accuracy ACC = 0.94, Sensitivity = 0.92, Specificity = 0.94, PPV = 0.80, NPV = 0.98, F_1_ score=0.86.

- In the testing datasets "Beijing HDxt" (subplot A) and the testing dataset "Guangzhou HDxt" (subplot B), the regression and classification performance were as follows.


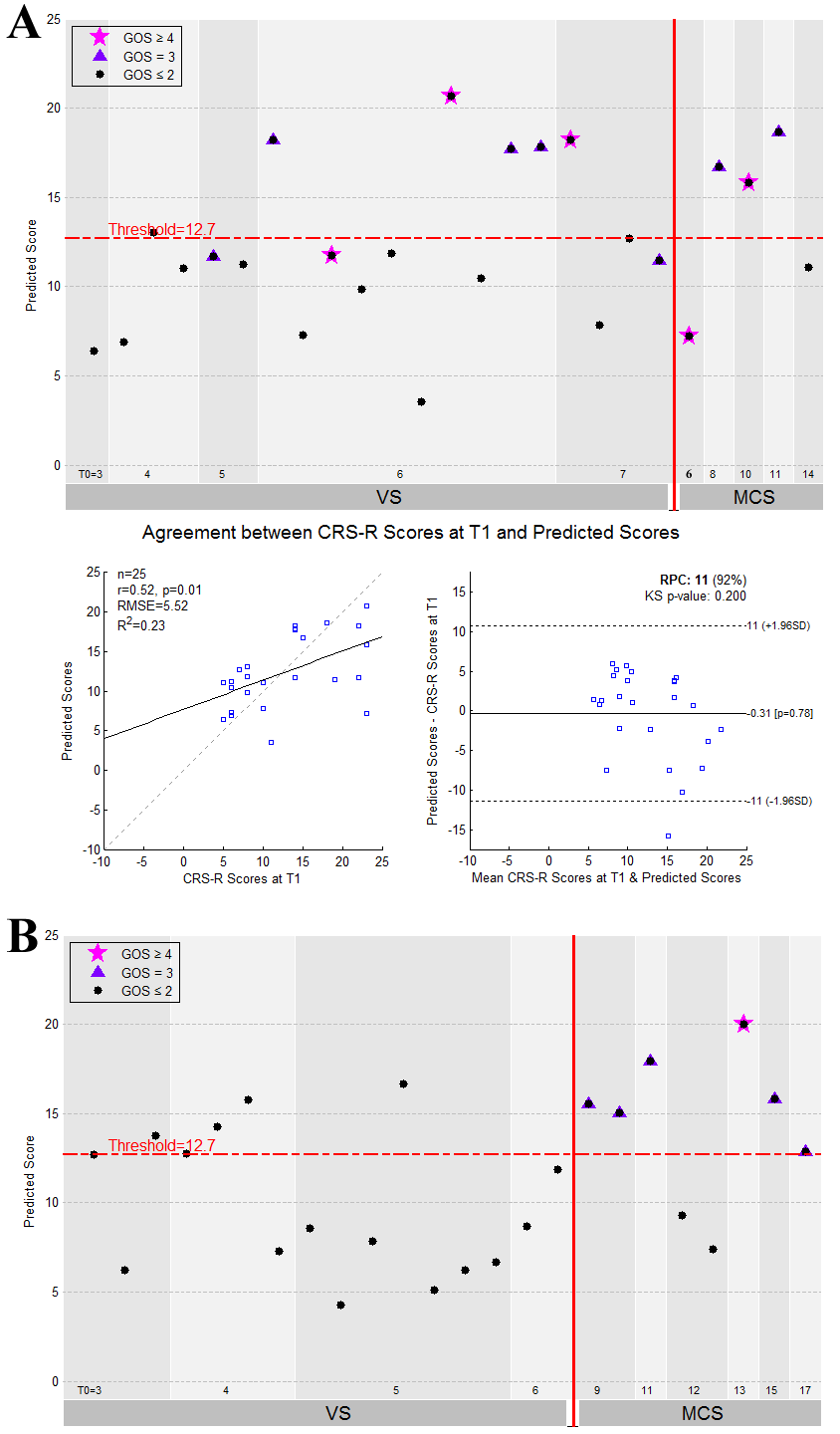


The testing dataset "Beijing HDxt": classification ACC = 0.80, Sensitivity = 0.67, Specificity = 0.92, PPV = 0.89, NPV = 0.75, F_1_ score=0.76; The testing dataset "Guangzhou HDxt" classification ACC = 0.79, Sensitivity = 1.00, Specificity = 0.72, PPV = 0.55, NPV = 1.00, F_1_ score=0.71.

(3). Comparison of the single-domain and combination models

We resampled with replacement from the training dataset "Beijing 750", and built a regression and classification model based on clinical features alone, neuroimaging features alone, or a combination of the two-domain features. We then tested the classification accuracy in the two testing datasets using the three predictive models. In this way, we obtained the distribution of the prediction accuracies using each of the three types of the models. Next, we used repeated measures ANOVA to test whether or not the performances of the three types of models were the same, as well as Ψ, the root-mean-square standardized effect, to report the effect sizes of the mean differences between them. The effect size analysis was carried out using the Matlab toolbox "Measures of Effect Size" (https://github.com/hhentschke/measures-of-effect-size-toolbox). The results in the two testing datasets are as follows.

- the testing dataset "Beijing HDxt"

| Imaging+clincial feature | Imaging feature | Clinical feature |
| --- | --- | --- |
| 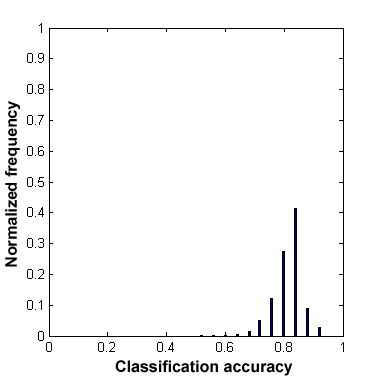 | 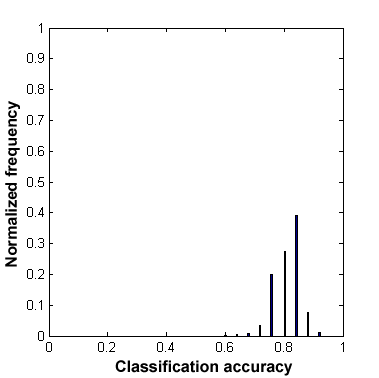 | 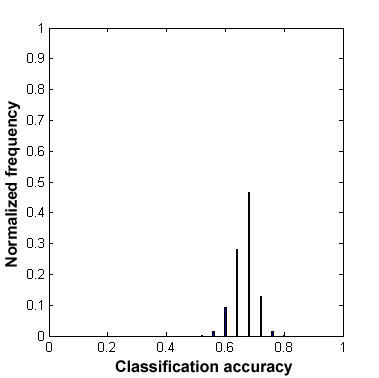 |
| Mean = 0.815  Standard deviation = 0.050 | Mean = 0.811  Standard deviation =0.044 | Mean = 0.666  Standard deviation =0.037 |

Using repeated measures ANOVA, we found that there were significant differences between the mean of the classification accuracies using the three types of models (p<0.001). We subsequently conducted pairwise comparisons, and found that there was a significant difference between the combination model and the model that used imaging features alone (paired sample t-test, p=0.001) or clinical features alone (paired sample t-test, p<0.001). We also found that there was significant difference between the model using imaging features alone and the model using clinical features alone (paired sample t-test, p<0.001).

Using effect size analysis, we found that there was a mean difference of Ψ=0.004 ( 95% confidence intervals [0.002, 0.007]) between the combination model and the model using imaging features alone, and Ψ=0.149 ( 95% confidence intervals [0.147, 0.152]) between the combination method and the model using clinical features alone. We also found that there was a mean difference of Ψ=0.145 ( 95% confidence intervals [0.142, 0.147]) between the methods using imaging features alone and clinical features alone.

- the testing dataset "Guangzhou HDxt"

| Imaging+clincial feature | Imaging feature | Clinical feature |
| --- | --- | --- |
| 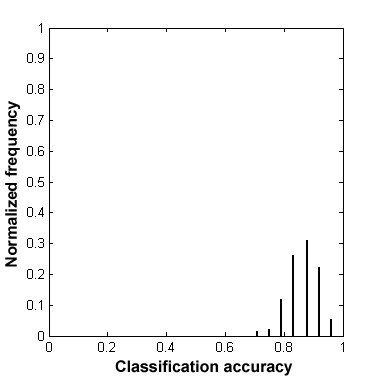 | 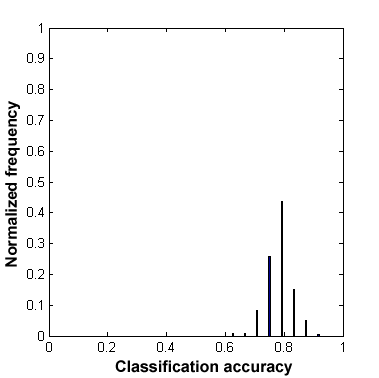 | 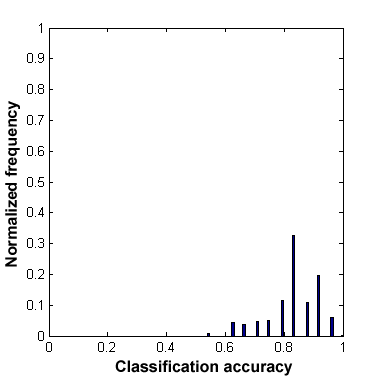 |
| Mean = 0.863  Standard deviation = 0.051 | Mean = 0.783  Standard deviation =0.044 | Mean = 0.829  Standard deviation =0.086 |

Using repeated measures ANOVA, we found that there were significant differences between the mean of the classification accuracies using the three types of models (p<0.001). We subsequently conduct pairwise comparisons, and found that there was a significant difference between the combination model and the model using imaging features alone (paired sample t-test, p<0.001) or clinical features alone (paired sample t-test, p<0.001). We also found that there was a significant difference between the model using imaging features alone and that only using clinical features (paired sample t-test, p<0.001).

Using effect size analysis, we found that there was a mean difference of Ψ=0.080( 95% confidence intervals [0.076, 0.084]) between the combination model and the model using imaging features alone, and a mean difference of Ψ=0.034 ( 95% confidence intervals [0.028, 0.040]) between the combination model and the model using clinical features alone. We also found that there was a mean difference of Ψ= -0.046 ( 95% confidence intervals [-0.053, -0.040]) between the model using only imaging features and that using only clinical features.

- Summary

Firstly, in both the two testing datasets, the combination of imaging and clinical features demonstrated better performances than using imaging features alone or clinical features alone. Secondly, the mean differences between the models using neuroimaging features alone or clinical features alone were reversed in the two testing datasets, which suggested that the two testing datasets were heterogeneous and that it is necessary to integrate different information to improve prognostication for DOC.
