# Supplementary material for: Prognostication of chronic disorders of consciousness using brain functional networks and clinical characteristics
Source: eLife. 2018 Aug 14;7:e36173. doi: 10.7554/eLife.36173 (PMC6145856; doi:10.7554/eLife.36173)
Supplement: Transparent reporting form [file elife-36173-transrepform.docx]

***eLife’s* transparent reporting form**

We encourage authors to provide detailed information *within their submission* to facilitate the interpretation and replication of experiments.Authors can upload supporting documentation to indicate the use of appropriate reporting guidelines for health-related research (see [EQUATOR Network](http://www.equator-network.org/%20)), life science research (see the [BioSharing Information Resource](https://biosharing.org/)), or the [ARRIVE guidelines](http://www.plosbiology.org/article/info:doi/10.1371/journal.pbio.1000412) for reporting work involving animal research.Where applicable, authors should refer to any relevant reporting standards documents in this form.

If you have any questions, please consult our Journal Policies and/or contact us:[editorial@elifesciences.org](mailto:editorial@elifesciences.org).

**Sample-size estimation**

- You should state whether an appropriate sample size was computed when the study was being designed
- You should state the statistical method of sample size computation and any required assumptions
- If no explicit power analysis was used, you should describe how you decided what sample (replicate) size (number) to use

Please outline where this information can be found within the submission (e.g., sections or figure legends), or explain why this information doesn’t apply to your submission:

We did not perform pre-study power calculations, however all the data available according to the included and excluded criteria of this study was used. The subject information can be found in the section "Subjects" and Table 1. In addition, Appendix 1 includes anonymised demographic and clinical characteristics for each enrolled patient.

**Replicates**

- You should report how often each experiment was performed
- You should include a definition of biological versus technical replication
- The data obtained should be provided and sufficient information should be provided to indicate the number of independent biological and/or technical replicates
- If you encountered any outliers, you should describe how these were handled
- Criteria for exclusion/inclusion of data should be clearly stated
- High-throughput sequence data should be uploaded before submission, with a private link for reviewers provided (these are available from both GEO and Array Express)

Please outline where this information can be found within the submission (e.g., sections or figure legends), or explain why this information doesn’t apply to your submission:

The out-of-bag estimation was provided to measure the generalizability of our method in the training dataset. In addition, the performance of the prediction model on two unseen testing datasets was provided. These results can be found in the section of "Results".

Criteria for exclusion/inclusion of data can be found in the section of "Subjects".

**Statistical reporting**

- Statistical analysis methods should be described and justified
- Raw data should be presented in figures whenever informative to do so (typically when N per group is less than 10)
- For each experiment, you should identify the statistical tests used, exact values of N, definitions of center, methods of multiple test correction, and dispersion and precision measures (e.g., mean, median, SD, SEM, confidence intervals; and, for the major substantive results, a measure of effect size (e.g., Pearson's r, Cohen's d)
- Report exact p-values wherever possible alongside the summary statistics and 95% confidence intervals. These should be reported for all key questions and not only when the p-value is less than 0.05.

Please outline where this information can be found within the submission (e.g., sections or figure legends), or explain why this information doesn’t apply to your submission:

The prognostic regression model was internally validated using bootstrap, and the out-of-bag estimation was provided to measure the generalizability of our method in the training dataset. This information can be found in the section of "Internal validation of model".

The classifier performance in two testing datasets were statistically tested with permutation test in the section of "Model external validation ".

The comparisons of the single-domain and combination models were carried out with repeated ANOVA and the effect sizes were reported. This information can be found in the section "Comparison of the single-domain and combination models ".

(For large datasets, or papers with a very large number of statistical tests, you may upload a single table file with tests, Ns, etc., with reference to sections in the manuscript.)

**Group allocation**

- Indicate how samples were allocated into experimental groups (in the case of clinical studies, please specify allocation to treatment method);if randomization was used, please also state if restricted randomization was applied
- Indicate if masking was used during group allocation, data collection and/or data analysis

Please outline where this information can be found within the submission (e.g., sections or figure legends), or explain why this information doesn’t apply to your submission:

This study subdivided the DOC patients according to their Glasgow Outcome Scale (GOS) score, i.e. "consciousness recovery" (GOS≥3) versus "consciousness non-recovery" (GOS≤2). This information can be found in the section of "Subjects".

**Additional data files (“source data”)**

- We encourage you to upload relevant additional data files, such as numerical data that are represented as a graph in a figure, or as a summary table
- Where provided, these should be in the most useful format, and they can be uploaded as “Source data” files linked to a main figure or table
- Include model definition files including the full list of parameters used
- Include code used for data analysis (e.g., R, MatLab)
- Avoid stating that data files are “available upon request”

Please indicate the figures or tables for which source data files have been provided:

The predicted score for each patient involved in our study was provided in the cell of "Predicted score" in Appendix 1.

We have made the analysis pipeline, including fMRI preprocessing, feature calculation and extraction, regression and classification, and the results visualization publicly available. Anyone is welcome to download it from GitHub (https://github.com/realmsong504/pDOC).
